# Serological Biomarkers in Individuals with Interstitial Lung Disease after SARS-CoV-2 Infection and Association with Post-COVID-19 Symptoms

**DOI:** 10.3390/pathogens13080641

**Published:** 2024-07-31

**Authors:** Paula Parás-Bravo, César Fernández-de-las-Peñas, Diego Ferrer-Pargada, Sheila Izquierdo-Cuervo, Luis M. Fernández-Cacho, José M. Cifrián-Martínez, Patricia Druet-Toquero, Oscar Pellicer-Valero, Manuel Herrero-Montes

**Affiliations:** 1 Departamento de Enfermería, Universidad de Cantabria, 39005 Santander, Spain; paula.paras@unican.es (P.P.-B.); luismanuel.fernandez@unican.es (L.M.F.-C.); manuel.herrero@unican.es (M.H.-M.); 2 Grupo de Investigación en Enfermería, Instituto de Investigación Sanitaria Valdecilla (IDIVAL), 39011 Santander, Spain; 3 Department of Physical Therapy, Occupational Therapy, Physical Medicine and Rehabilitation, Universidad Rey Juan Carlos (URJC), 28922 Madrid, Spain; 4 Servicio de Neumología, Hospital Universitario Marqués de Valdecilla, 39008 Cantabria, Spain; diegojose.ferrer@scsalud.es (D.F.-P.); sheila.izquierdo@scsalud.es (S.I.-C.); josemanuel.cifrian@scsalud.es (J.M.C.-M.); patricia.druet@scsalud.es (P.D.-T.); 5 Image Processing Laboratory (IPL), Universitat de València, Parc Científic, Paterna, 46980 Valencia, Spain; oscar.pellicer@uv.es

**Keywords:** interstitial lung disease, SARS-CoV-2, post-COVID-19, serological biomarker, long COVID

## Abstract

Patients with interstitial lung disease (ILD) represent a vulnerable population against an acute SARS-CoV-2 infection. It has been observed that up to 80% of patients with ILD can develop post-COVID-19 symptomatology one year after. This secondary analysis aimed to, 1, compare serological biomarkers before and after surpassing a SARS-CoV-2 infection in individuals with interstitial lung disease (ILD) and, 2, to compare serological biomarkers between ILD patients who develop and those who do not develop post-COVID-19 symptoms. Seventy-six patients with ILD (40.4% women, age: 69, SD: 10.5 years) who survived a SARS-CoV-2 infection participated. High-resolution computerized tomography (CT) of the lungs, two pulmonary function tests (forced vital capacity (FVC) and diffusion value of carbon monoxide (DLCO)) and fourteen serological biomarkers were collected before and after SARS-CoV-2 infection. Participants were asked for the presence of post-COVID-19 symptomatology a mean of twelve (SD: eight) months after infection. Sixty patients (79%) showed post-COVID-19 symptoms (mean: 3.5, SD 1.1), with fatigue (68.4%), dyspnea (31.5%), and concentration loss (27.6%) being the most prevalent. Creatine phosphokinase (CPK) was the only biomarker showing differences in our study. In fact, CPK levels were higher after the acute SARS-CoV-2 infection (mean difference: 41.0, 95%CI 10.1 to 71.8, *p* = 0.03) when compared to before the infection. Thus, CPK levels were also higher in ILD patients with post-COVID-19 fatigue (mean difference: 69.7, 95%CI 12.7 to 126.7, *p* = 0.015) or with post-COVID-19 dyspnea (mean difference: 34.8, 95%CI 5.2 to 64.4, *p* = 0.025) than those patients without these post-COVID-19 symptoms. No significant changes in CT or functional pulmonary tests were observed after COVID-19 in patients with ILD. In conclusion, patients with ILD exhibited an increase in CPK levels after SARS-CoV-2 infection, albeit no changes in other serological biomarkers were identified. Similarly, the presence of post-COVID-19 fatigue or dyspnea was also associated with higher CPK levels in ILD patients. Studies investigating long COVID mechanisms in vulnerable populations such as ILD are needed.

## 1. Introduction

Chronic respiratory diseases are still considered one of the leading causes of death and disability within the general population [1]. Interstitial lung disease (ILD) consists of a group of heterogeneous respiratory disorders sharing radiographic manifestations and featuring bothersome symptoms such as dyspnea and fatigue [2].

The estimated prevalence of ILD has been found to range from 6.3 to 71 cases per 100,000 people [3]. The Global Burden of Disease Study has reported that the estimated prevalence and incidence rates of ILD have increased by 9.4% (6.1–12.9%) and 14.1% (11.1–17.3%), respectively, from 1990 to 2019 [1]. Thus, health-related quality of life is affected in patients with ILD, particularly at physical levels [4]; however, these patients can also exhibit mood disorders, e.g., depression or anxiety [5].

In 2020, the world experienced the most dramatic healthcare situation of the century due to the spread of the coronavirus disease, 2019 (COVID-19). The COVID-19 outbreak resulted in a staggering 776 million confirmed cases and more than 7 million related deaths globally [6]. Individuals suffering from respiratory conditions are considered vulnerable groups more susceptible to severe acute respiratory syndrome coronavirus 2 (SARS-CoV-2), the agent causing COVID-19 and its sequelae. Patients with ILD, as a vulnerable group, exhibit a higher risk of infection by SARS-CoV-2 [7], although ILD has not been found to be a risk factor for developing COVID-19-associated pneumonia [8].

Patients with ILD who experience COVID-19 can exhibit a worsening of their pulmonary fibrosis, which can be associated with a significant decline in their clinical status [9]. This decline in their clinical status can be related to a worsening of their respiratory disease, i.e., ILD, but it can also be related to the development of new symptomatology. In fact, the presence of new symptoms after an acute SARS-CoV-2 infection has been called long COVID [10] or post-COVID-19 condition [11]. During the last few years, more than 100 long-lasting post-COVID-19 symptoms affecting multiple (e.g., cardiovascular, respiratory, neurological, or musculoskeletal) systems have been attributed to COVID-19 [12], and they can persist for several months or years after the infection [13]. In fact, a recent meta-analysis reported that 25–30% of subjects who survived COVID-19 exhibit post-COVID-19 symptomatology up to two years after the initial infection [14].

Data about the prevalence of post-COVID-19 symptomatology in vulnerable populations are scarce [15]. We have previously observed that 80% of individuals with ILD who survived COVID-19 exhibit long-lasting post-COVID-19 symptoms one year after [16]. Post-COVID-19 fatigue, dyspnea, and concentration loss were the most prevalent symptoms in this population (68.4%, 31.5%, 27.6%, respectively) [16]. Based on these data, the prevalence of long-lasting post-COVID-19 symptomatology in patients with ILD is higher than the general population [14]. Accordingly, a better understanding of the potential underlying mechanisms specifically seen in patients with ILD could help inform management of post-COVID-19 symptomatology in this population.

The role of serological biomarkers at the acute phase of a SARS-CoV-2 infection has received particular attention to predict the risk of severe COVID-19. It has been seen that severe COVID-19 is characterized by higher levels of some inflammatory biomarkers such as C-reactive protein (CRP), erythrocyte sedimentation rate (ESR), procalcitonin (PCT), and interleukin 6 (IL-6) when compared with mild-to-moderate COVID-19 [17]. Nevertheless, the role of serological biomarkers in the development of long-lasting post-COVID-19 symptoms has been less investigated and with heterogeneous study designs. For instance, most studies investigating serological biomarkers at the acute COVID-19 phase did not observe significant associations with post-COVID-19 symptoms such as fatigue [18,19], dyspnea [20], or pain [21]. Other studies have investigated these serological biomarkers at the post-acute COVID-19 phase, that is, when post-COVID-19 symptomatology is present. The meta-analysis by Yoing et al. identified higher levels of CRP, lactate dehydrogenase (LDH), D-dimer, and a higher leucocyte count in individuals with post-COVID-19 symptoms when compared with COVID-19 survivors without symptoms [22]. However, they advised that serological biomarker levels could be post-COVID-19 symptom-specific [22]. This hypothesis is supported by Udeh et al., who reported that LDH levels are increased in individuals exhibiting respiratory post-COVID-19 symptoms, but not in those subjects with cardiac, neurological, or gastrointestinal post-COVID-19 symptoms [23]. No study has yet to investigate serological biomarker levels at the post-acute COVID-19 phase in people with ILD. Additionally, most previously published studies did not report data on serological biomarkers before SARS-CoV-2 infection [22,23].

We present here a secondary analysis of our cohort of patients with ILD [16], evaluating serological biomarkers at the post-acute COVID-19 phase. Therefore, the objectives of the current study are as follows: 1, to compare serological biomarkers before and after an acute SARS-CoV-2 infection in individuals with ILD; and 2, to compare serological biomarkers between patients with ILD who developed post-COVID-19 symptoms and those who did not develop post-COVID-19 symptomatology.

## 2. Methods

### 2.1. Study Design

A cross-sectional cohort study with a repeated measures design using pre-COVID-19 previously collected data was conducted according to the Strengthening the Reporting of Observational Studies in Epidemiology (STROBE) guidelines [24]. The Institutional Research Ethics Committee of the Hospital de Valdecilla Cantabria, Spain (code 2022.265), evaluated and approved the study design. Participants provided their written informed consent prior to their inclusion.

### 2.2. Participants

As previously described [16], consecutive individuals with ILD recruited from a specific unit on pulmonary fibrosis at the Hospital Universitario Marqués de Valdecilla, an urban hospital in Santander, Spain, were invited to participate in the study. Eligible participants fulfilled the American Thoracic Society and European Respiratory Society criteria [25]. The diagnosis of ILD was based on high-resolution computerized tomography (CT) and/or surgical transbronchial biopsies of the lungs. Participants were classified as follows [16]: 1, idiopathic ILD (i.e., idiopathic pulmonary fibrosis or idiopathic non-specific interstitial pneumonia); 2, autoimmune-related ILD; 3, exposure-related ILD; or 4, ILD caused by sarcoidosis [26]. To be included, patients must have reported a SARS-CoV-2 infection (ICD-10 code) diagnosed by a real-time reverse transcription-polymerase chain reaction assay of nasopharyngeal/oral swab samples.

### 2.3. Data Collection

The current study was a mixed design including data collected before the COVID-19 pandemic (retrospectively) and data after infection (prospectively). All patients with ILD attending the specific pulmonary fibrosis unit had an annual routine medical appointment for assessing their evolution. This appointment includes the following: 1, high-resolution lung CT; 2, two pulmonary function tests assessed with a Jaeger Carefusion MastersCreen combi model (Hospital Hispani SL, Madrid, Spain): forced vital capacity (FVC) and the diffusion value of carbon monoxide (DLCO) according to the American Thoracic Society standards [27]; and 3, extraction of a blood sample for assessing serological biomarkers (see Section 2.4).

As previously described [16], patients with ILD who had survived COVID-19 were systematically asked for the presence of symptoms that appeared after infection and that they attributed to SARS-CoV-2. Due to the potential overlap between ILD-associated symptoms and respiratory post-COVID-19 symptoms, we specifically looked for the following: 1, new symptoms that appeared post-COVID-19 that the patient did not experience before; or, 2, a clear worsening of any symptom commonly experienced by any ILD patient, e.g., dyspnea, after the infection. In this secondary analysis, we considered the presence of the three most prevalent post-COVID-19 symptoms reported by this cohort of patients with ILD [16]: fatigue, dyspnea, and concentration loss.

### 2.4. Serological Biomarkers

The following serological biomarkers are routinely collected in blood samples extracted in the pulmonary fibrosis unit at the Hospital de Valdecilla Cantabria, Spain: hemoglobin, lymphocyte count, neutrophil count, platelet count, glucose, albumin, sodium, potassium, alanine transaminase (ALT), aspartate transaminase (AST), lactate dehydrogenase (LDH), creatine phosphokinase (CPK), and D-dimer.

### 2.5. Statistical Analysis

Statistical analyses were conducted with Stata Statistical Software version 16.1 (StataCorp LP, College Station, TX, USA). Means (standard deviation, SD) or number (percentage) are presented for quantitative and categorical variables, respectively. The Kolmogorov–Smirnov test revealed that all quantitative data exhibited a normal distribution. Missing values were managed by using imputation. McNemar’s chi-squared test and paired Student t-tests were conducted to compare proportions and means before and after SARS-CoV-2 infection (first objective), whereas unpaired Student t-tests were used to compare means between patients with and without post-COVID-19 fatigue, dyspnea, or concentration loss. The level of significance was set a priori at *p* < 0.05, but all tests were corrected (Holm–Bonferroni correction).

## 3. Results

### 3.1. Participants

As previously reported [16], from a total of 173 patients with ILD who visited the pulmonary fibrosis unit from 1 November 2022 to 1 June 2023, 76 (44%) had survived COVID-19 and agreed to participate in the current study. All patients had received two doses (completed vaccination), and 70% had received a booster dose of BNT162b2 vaccine (“Pfizer/BioNTech”) before SARS-CoV-2 infection [16].

### 3.2. Symptoms Associated with SARS-CoV-2 Infection

Patients with ILD who survived an acute SARS-CoV-2 infection were evaluated a mean of twelve (SD: eight) months after the infection. At the time of examination, 60/76 (79%) reported post-COVID-19 symptomatology (mean number of post-COVID-19 symptoms: 3.5, SD 1.1).

We should note the fluctuating nature of symptoms associated with SARS-CoV-2 at the acute COVID-19 and post-COVID-19 phases: 1, a COVID-19-associated onset symptom appears at the acute COVID-19 phase and persists as a post-COVID-19 symptom; 2, a COVID-19-associated onset symptom appears at the acute COVID-19 phase but disappears when the acute phase is over; 3, a symptom that was not experienced at the acute COVID-19 phase is experienced as a post-COVID-19 symptom. Table 1 details COVID-19 onset symptoms and the post-COVID-19 symptomatology of the cohort [16].

In our cohort of patients with ILD, of the 56 patients who experienced fatigue as a COVID-19-associated onset symptom, 47/56 (84%) experienced it as a persistent post-COVID-19 symptom, whereas 5/52 (9.5%) of patients developed fatigue as a new post-COVID-19 symptom. Thus, of the 26 patients with ILD who experienced dyspnea as a COVID-19-associated onset symptom, 18/26 (69%) experienced it as a persistent post-COVID-19 symptom, whereas 6/24 (25%) developed dyspnea as a new post-COVID-19 symptom.

As expected, some COVID-19-associated onset symptoms such as fever or cough were present only at the acute phase of the infection, but they were not experienced as post-COVID-19 symptoms. Finally, concentration loss was only experienced as a post-COVID-19 symptom but not as a COVID-19-associated onset symptom (Table 1).

### 3.3. Changes in CT Scan and Pulmonary Function Tests

Data were collected before (mean: 11, SD: 7 months) and after (mean: 11; SD: 8 months) SARS-CoV-2 infection. According to the CT, 39% (*n* = 30/76) of the patients exhibited idiopathic ILD, 48% (*n* = 34/76) exhibited autoimmune-related ILD, and the remaining 12/76 (16%) patients exhibited exposure-related ILD. The CT scan after COVID-19 did not reveal any significant change in most patients, and just a small progression in four patients.

Similarly, no significant changes (both, *p* > 0.7) in pulmonary function tests before (FVC, mean: 79%, SD: 22.8%; DLCO, mean: 66.9%, SD: 22.3%) and after (FVC, mean: 79.2, SD: 22.2%; DLCO, mean: 66.2%, SD: 23.2%) the infection were observed.

### 3.4. Serological Biomarkers before and after SARS-CoV-2 Infection

Table 2 details data of serological markers before and after acute SARS-CoV-2 infection. Overall, no significant changes in any of the serological biomarkers analyzed before and after COVID-19 were identified, except for CPK levels (*p* = 0.03): CPK levels were significantly higher after the acute SARS-CoV-2 infection (mean difference: 41.0, 95%CI 10.1 to 71.8).

### 3.5. Serological Biomarkers According to the Presence or Absence of Post-COVID-19 Symptoms

Similarly, no significant differences in the serological biomarkers analyzed were found between ILD patients reporting or not reporting post-COVID-19 fatigue, except for differences in CPK levels (*p* = 0.015, Table 3) and lymphocyte count: CPK levels (mean difference: 69.7, 95%CI 12.7 to 126.7, *p* = 0.015) and lymphocyte count (mean difference: 0.4, 95%CI 0.1 to 0.7, *p* = 0.037) were significantly higher in ILD patients with post-COVID-19 fatigue than in those without post-COVID-19 fatigue (Table 3).

Furthermore, serological biomarkers were not significantly different between ILD patients with and without post-COVID-19 dyspnea, except for CPK levels (*p* = 0.025, Table 4): CPK levels were significantly higher (mean difference: 34.8, 95%CI 5.2 to 64.4) in ILD patients who developed post-COVID-19 dyspnea when compared with those without post-COVID-19 fatigue (Table 4).

Finally, no differences in any serological biomarker analyzed were identified according to the presence or absence of post-COVID-19 concentration loss (Table 5).

## 4. Discussion

This study investigated the role of several serological biomarkers after COVID-19 in a vulnerable population such as ILD. The results identified an increase in CPK levels, suggestive of muscle damage, one year after an infection by SARS-CoV-2 and also in those patients who developed post-COVID-19 fatigue or post-COVID-19 dyspnea, but not in those reporting post-COVID-19 concentration loss.

As previously reported [16], patients with ILD exhibited a higher prevalence rate of post-COVID-19 fatigue (68.4%) and post-COVID-19 dyspnea (31.5%) than the general population [13,14]. It has been found that patients with ILD can experience a worsening of their pulmonary fibrosis after surviving an acute SARS-CoV-2 infection [9]. In addition, it has also been suggested that the pathophysiology of pulmonary fibrosis seen in COVID-19 survivors with post-COVID-19 symptomatology shares common mechanisms with ILD [28]. In fact, the term post-COVID-19 ILD has been proposed, since up to 15% of COVID-19 patients exhibit pulmonary fibrosis processes similar to patients with idiopathic ILD [29]. However, we did not observe this finding in our sample of patients with ILD, since no significant changes were observed in pulmonary fibrosis throughout CT scans before and after COVID-19. In fact, we also did not find significant changes in functional pulmonary tests before and after COVID-19 in our sample of ILD patients.

The underlying mechanisms explaining post-COVID-19 fatigue and dyspnea in people who survived an acute SARS-CoV-2 infection include an exaggerated systemic immune response, prolonged inflammation, an atypical response of mast cells, hypercoagulability, and lung damage [30,31]. It is important to note that no specific serological biomarker has yet been associated with the post-COVID-19 condition to date. Yoing et al. found higher levels of several serological biomarkers (CRP, LDH, and D-dimer) in COVID-19 survivors with post-COVID-19 symptomatology; however, it seems that this association can be symptom-specific [22]. For instance, Udeh et al. reported that higher LDH levels were seen in COVID-19 survivors with respiratory post-COVID-19 symptoms, but not in those with other types of post-COVID-19 symptoms [23]. Although LDH levels were slightly higher in ILD patients reporting post-COVID-19 fatigue or dyspnea, differences were not significant in our study, probably due to the sample size.

We also observed higher CPK levels in our sample of ILD patients with respiratory post-COVID-19 symptoms. Thus, CPK levels were also higher in the total sample of ILD patients after the infection as compared with before (Table 2). Bhalla et al. observed elevated CPK levels at the acute phase in individuals with severe COVID-19 [32]. In fact, hypercalcemia, i.e., higher CPK levels, has been associated with respiratory failure during the acute COVID-19 phase [33]. Accordingly, an elevation in CPK levels after SARS-CoV-2 infection would support potential further lung damage in patients with ILD, which could explain a worsening of pulmonary fibrosis [9]. In addition, this worsening of pulmonary fibrosis could also be associated with the development of “de novo” respiratory symptoms such as post-COVID-19 fatigue or dyspnea after the infection. However, we did not find a worsening of pulmonary fibrosis in our sample of patients with ILD, as reflected by the CT scans. Finally, although higher CPK levels are usually identified with muscular damage, Friedman et al. did not observe an association between elevated levels of the biomarker with skeletal muscle symptoms/signs or with other laboratory markers [34], supporting the respiratory repercussion of high levels of CPK.

Another result of the current study was that ILD patients reporting post-COVID-19 fatigue, but not dyspnea, showed a higher lymphocyte count than those who did not report post-COVID-19 fatigue. The findings suggest a significant immune response against SARS-CoV-2 infection in ILD patients developing post-COVID-19 fatigue [35]. It is possible that an exaggerated immune response in this population would promote immune cell exhaustion, which has been proposed as a mechanism of the post-COVID-19 condition [36]. Nevertheless, it should be noted that the lymphocyte count was not significantly different in the overall sample one year after the infection (Table 2), supporting the hypothesis that this immune cell exhaustion could be post-COVID-19 symptom-specific. Additionally, although the lymphocyte count in ILD patients suffering from post-COVID-19 fatigue (mean: 2.1, SD: 0.8) was higher, from a statistical viewpoint, than in those who did not develop this post-COVID-19 symptom (mean: 1.7, SD: 1.0), these counts fall within the normal range of lymphocytes; accordingly, further immunological studies are needed to confirm or refute the hypothesis of an exaggerated immune response.

Finally, although this is the first study investigating changes in different serological biomarkers in ILD patients who survived an acute SARS-CoV-2 infection, some limitations must be recognized. First, although we collected serological data before and after COVID-19 in a cohort of patients with ILD, data were based on medical records. In fact, among the fourteen serological biomarkers that we analyzed, CRP, which has been commonly investigated in COVID-19, was not included, since the routine assessment of these patients in our pulmonary fibrosis unit did not collect it. Second, the cross-sectional nature of the study did not permit an assessment of the evolution of these serological biomarkers. Thus, we did not collect serological biomarkers at the acute COVID-19 phase; accordingly, we do not know the role of serological biomarkers at the acute SARS-CoV-2 infection phase and if these biomarkers could help to identify ILD patients at a higher risk of developing post-COVID-19 symptoms. Third, we mainly collected self-reported post-COVID-19 symptoms. Although we specifically asked for the presence of symptoms appearing after the SARS-CoV-2 infection, it is possible that respiratory post-COVID-19 symptoms overlapped with some symptoms previously experienced due to ILD, since these patients also exhibit fatigue and dyspnea associated with the disease. Fourth, the limited sample size did not permit us to conduct sub-group analyses according to ILD type nor to evaluate sex differences. Female sex has been identified as a risk factor for post-COVID-19 conditions in the general population [37]. We do not currently know if female sex would also be a risk factor for post-COVID-19 conditions in vulnerable populations. Finally, we investigated the role of fourteen serological biomarkers within the three most prevalent post-COVID-19 symptoms experienced by our sample of patients with ILD. Future studies including large samples with a greater number of patients with other type of post-COVID-19 symptoms would help to clarify the role of these biomarkers in this population.

## 5. Conclusions

This study found an increase in CPK levels, suggestive of muscle damage, one year after COVID-19 in a sample of individuals with ILD. Thus, CPK levels were also higher in patients with ILD who developed post-COVID-19 fatigue or post-COVID-19 dyspnea, but not in those with post-COVID-19 concentration loss. Individuals with ILD reporting post-COVID-19 fatigue, but not dyspnea, showed a higher lymphocyte count than those who did not report post-COVID-19 fatigue. Finally, no significant changes in CT scans or functional pulmonary tests were observed after COVID-19 in patients with ILD.

## Figures and Tables

**Table 1:** Demographic data and COVID-19-associated data of patients with interstitial lung disease infected by SARS-CoV-2 (*n* = 76) [16].

Demographic Data	
Age (years)	69.0 ± 10.5
Sex (male/female)	45 (59.2%)/31 (40.8%)
Weight (kg)	77.5 ± 16.5
Height (cm)	168.0 ± 10.0
**COVID-19-associated onset symptoms**	
Fatigue, *n* (%)	56 (73.7%)
Muscle pain *n* (%)	54 (71.0%)
Cough, *n* (%)	53 (69.7%)
Fever, *n* (%)	44 (57.9%)
Headache, *n* (%)	38 (50.0%)
Throat pain, *n* (%)	33 (43.4%)
Dyspnea, *n* (%)	26 (34.2%)
Ageusia, *n* (%)	18 (23.7%)
Anosmia, *n* (%)	16 (21.0%)
Dizziness, *n* (%)	15 (19.7%)
Diarrhea, *n* (%)	11 (14.5%)
Vomiting, *n* (%)	5 (6.5%)
**Post-COVID-19 Symptoms**	
Fatigue, *n* (%)	52 (68.4%)
Dyspnea, *n* (%)	24 (31.5%)
Throat pain, *n* (%)	22 (28.9%)
Concentration loss, *n* (%)	21 (27.6%)
Brain Fog, *n* (%)	19 (25.0%)
Ageusia, *n* (%)	16 (21.0%)
Memory loss, *n* (%)	15 (19.7%)
Anosmia, *n* (%)	14 (18.4%)
Pain, *n* (%)	13 (17.1%)
Hair loss, *n* (%)	13 (17.1%)
Tachycardia, *n* (%)	11 (14.5%)
Skin rashes, *n* (%)	10 (13.3%)

**Table 2:** Serological biomarkers of patients with interstitial lung disease before and after SARS-CoV-2 infection.

Serological Biomarkers, Mean (SD)	Before COVID-19	After COVID-19	*p* Value
Lactate dehydrogenase (LDH, U/L)	238.5 (47.7)	237.0 (54.0)	0.829
Creatine phosphokinase (CPK, mcg/L) *	133.5 (82.9)	174.5 (46.5)	0.03 *
Leucocytes (×109/L)	7.5 (3.2)	7.6 (2.6)	0.603
Neutrophils (×109/L)	4.5 (1.7)	4.4 (1.8)	0.938
Lymphocytes (×109/L)	1.8 (0.9)	1.9 (0.8)	0.694
Alanine transaminase (ALT, U/L)	26.0 (12.2)	25.1 (13.7)	0.411
Aspartate transaminase (AST, U/L)	28.2 (11.4)	26.3 (9.5)	0.172
Glucose (mg/mL)	99.5 (24.6)	100.2 (36.4)	0.874
Hemoglobin (g/dL)	14.0 (1.5)	13.8 (1.55)	0.812
Platelets (×109/L)	226.65 (99.5)	227.0 (57.5)	0.973
D-dimer (ng/mL)	919.4 (914.05)	1040.65 (941.75)	0.238
Albumin (g/dL)	4.3 (0.35)	4.3 (0.3)	0.392
Sodium (mEq/L)	137.8 (3.9)	140.0 (2.1)	0.272
Potassium (mmol/L)	4.3 (0.45)	4.4 (0.4)	0.483

* Statistically significant differences between groups (*p* < 0.05).

**Table 3:** Serological biomarkers of patients with interstitial lung disease with and without fatigue as a post-COVID-19 symptom.

Serological Biomarkers, Mean (SD)	No Fatigue (*n* = 24)	Fatigue (*n* = 52)	*p* Value
Lactate dehydrogenase (LDH, U/L)	222.6 (47.9)	239.4 (53.2)	0.216
Creatine phosphokinase (CPK, mcg/L) *	112.2 (62.1)	181.9 (48.2)	0.015 *
Leucocytes (×109/L)	7.4 (2.7)	8.1 (3.1)	0.302
Neutrophils (×109/L)	4.0 (2.0)	3.9 (2.0)	0.905
Lymphocytes (×109/L) *	1.7 (1.0)	2.1 (0.8)	0.037 *
Alanine transaminase (ALT, U/L)	25.3 (16.2)	24.0 (12.1)	0.685
Aspartate transaminase (AST, U/L)	27.5 (9.9)	25.0 (9.0)	0.289
Glucose (mg/mL)	109.8 (56.1)	97.7 (26.2)	0.206
Hemoglobin (g/dL)	14.0 (1.1)	13.8 (1.65)	0.752
Platelets (×109/L)	229.65 (55.1)	227.8 (57.3)	0.827
D-dimer (ng/mL)	939.0 (684.5)	1095.5 (725.5)	0.380
Albumin (g/dL)	4.25 (0.25)	4.3 (0.3)	0.830
Sodium (mEq/L)	139.9 (2.3)	140.1 (2.1)	0.636
Potassium (mmol/L)	4.5 (0.4)	4.4 (0.4)	0.718

* Statistically significant differences between groups (*p* < 0.05).

**Table 4:** Serological biomarkers of patients with interstitial lung disease with and without dyspnea as a post-COVID-19 symptom.

Serological Biomarkers, Mean (SD)	No Dyspnea (*n* = 52)	Dyspnea (*n* = 24)	*p* Value
Lactate dehydrogenase (LDH, U/L)	229.4 (51.6)	243.7 (52.2)	0.272
Creatine phosphokinase (CPK, mcg/L) *	117.4 (68.3)	152.2 (55.8)	0.025 *
Leucocytes (×109/L)	7.8 (3.1)	7.9 (2.8)	0.769
Neutrophils (×109/L)	4.1 (2.0)	3.8 (1.9)	0.338
Lymphocytes (×109/L)	1.9 (0.85)	1.9 (1.1)	0.918
Alanine transaminase (ALT, U/L)	24.6 (14.0)	23.9 (12.5)	0.623
Aspartate transaminase (AST, U/L)	25.7 (8.7)	25.8 (10.4)	0.879
Glucose (mg/mL)	106.1 (45.3)	92.6 (24.4)	0.144
Hemoglobin (g/dL)	13.8 (1.5)	13.9 (1.5)	0.892
Platelets (×109/L)	223.8 (52.3)	236.3 (63.2)	0.464
D-dimer (ng/mL)	961.0 (684.7)	1080.0 (833.6)	0.175
Albumin (g/dL)	4.3 (0.2)	4.2 (0.4)	0.877
Sodium (mEq/L)	139.8 (2.4)	140.4 (1.9)	0.796
Potassium (mmol/L)	4.45 (0.4)	4.3 (0.35)	0.693

* Statistically significant differences between groups (*p* < 0.05).

**Table 5:** Serological biomarkers of patients with interstitial lung disease with and without concentration loss as a post-COVID-19 symptom.

Serological Biomarkers, Mean (SD)	No Concentration Loss (*n* = 55)	Concentration Loss (*n* = 21)	*p* Value
Lactate dehydrogenase (LDH, U/L)	229.6 (51.7)	247.7 (51.2)	0.196
Creatine phosphokinase (CPK, mcg/L)	118.0 (68.5)	144.2 (65.9)	0.140
Leucocytes (×109/L)	8.0 (3.1)	7.5 (2.8)	0.451
Neutrophils (×109/L)	4.2 (1.9)	3.7 (1.9)	0.262
Lymphocytes (×109/L)	1.9 (0.8)	1.8 (1.2)	0.818
Alanine transaminase (ALT, U/L)	25.1 (14.1)	22.5 (11.4)	0.451
Aspartate transaminase (AST, U/L)	25.6 (8.7)	26.0 (10.8)	0.898
Glucose (mg/mL)	104.5 (43.6)	93.6 (14.3)	0.269
Hemoglobin (g/dL)	14.0 (1.4)	13.5 (1.6)	0.593
Platelets (×109/L)	223.1 (53.1)	240.9 (63.1)	0.223
D-dimer (ng/mL)	989.0 (752.5)	1033.0 (715.8)	0.363
Albumin (g/dL)	4.5 (0.2)	4.25 (0.4)	0.768
Sodium (mEq/L)	139.8 (2.5)	140.6 (1.7)	0.825
Potassium (mmol/L)	4.5 (0.4)	4.25 (0.35)	0.283

## Data Availability

All data relevant to the study are included in the article.
